# The *in vitro* GcMAF effects on endocannabinoid system transcriptionomics, receptor formation, and cell activity of autism-derived macrophages

**DOI:** 10.1186/1742-2094-11-78

**Published:** 2014-04-17

**Authors:** Dario Siniscalco, James Jeffrey Bradstreet, Alessandra Cirillo, Nicola Antonucci

**Affiliations:** 1 Department of Experimental Medicine, Second University of Naples, via S. Maria di Costantinopoli, 16 - 80138 Naples, Italy; 2 Centre for Autism - La Forza del Silenzio, Caserta 81036, Italy; 3 Cancellautismo- no profit association for autism care, Florence 50132, Italy; 4 Brain Treatment Center of Atlanta, Atlanta, GA 30518, USA; 5 Western University of Health Science, 309 E. Second St., Pomona, CA 91766, USA; 6 Institute of Bioscience and Bioresources, National Research Council of Italy, Naples 80128, Italy; 7 Biomedical Center for Autism Research and Treatment, Bari 70126, Italy

**Keywords:** GcMAF, Endocannabinoids, Gene expression, Macrophages, Autism

## Abstract

**Background:**

Immune system dysregulation is well-recognized in autism and thought to be part of the etiology of this disorder. The endocannabinoid system is a key regulator of the immune system via the cannabinoid receptor type 2 (CB2R) which is highly expressed on macrophages and microglial cells. We have previously published significant differences in peripheral blood mononuclear cell *CB2R* gene expression in the autism population. The use of the Gc protein-derived Macrophage Activating Factor (GcMAF), an endogenous glycosylated vitamin D binding protein responsible for macrophage cell activation has demonstrated positive effects in the treatment of autistic children. In this current study, we investigated the *in vitro* effects of GcMAF treatment on the endocannabinoid system gene expression, as well as cellular activation in blood monocyte-derived macrophages (BMDMs) from autistic patients compared to age-matched healthy developing controls.

**Methods:**

To achieve these goals, we used biomolecular, biochemical and immunocytochemical methods.

**Results:**

GcMAF treatment was able to normalize the observed differences in dysregulated gene expression of the endocannabinoid system of the autism group. GcMAF also down-regulated the over-activation of BMDMs from autistic children.

**Conclusions:**

This study presents the first observations of GcMAF effects on the transcriptionomics of the endocannabinoid system and expression of CB2R protein. These data point to a potential nexus between endocannabinoids, vitamin D and its transporter proteins, and the immune dysregulations observed with autism.

## Introduction

Autism and autism spectrum disorders (ASDs) are complex heterogeneous neurodevelopmental conditions [1], arising from the interaction of genetic and environmental factors [2]. The established symptom categories include dysfunctions in communication skills and social interactions, combined with repetitive, restrictive and stereotypic verbal and non-verbal behaviors. Despite extensive research efforts, the etiopathologies of ASDs remain inadequately understood [3-5]. Early inflammatory processes, including maternal-fetal immune interactions and resultant immunological dysfunctions have been proposed as potential mechanisms [6-9]. The prevailing hypothesis is that some combination of immune factors including maternally-developed antibodies to fetal brain, prime microglia in such a way as to preclude their normal functions of directing neuronal migration and pruning [10,11].

The functional role of Vitamin D in the central nervous system has recently been reviewed and includes neurogenesis, neuroplasticity and a neuroprotection [12]. Vitamin D deficiency has been a demonstrated cause of recurrent pregnancy loss and supplementation with D3 significantly reduces IFN-γ and TNF-α secretion from natural killer (NK) cells [13]. There is a complex interaction between vitamin D and polymorphisms of the vitamin D receptor (VDR) and both the risk of autoimmunity and the responsiveness to vitamin D supplementation [14]. In autism, vitamin D deficiency in pregnancy or early childhood appears to contribute significantly to risk [15].

Potentially related to these processes are the recent observations of elevated N-acetylgalactosaminidase (Nagalase) levels in the blood of children with ASDs [16]. Nagalase is an enzyme that catalyzes the deglycosylation of the Gc protein also known as vitamin D3 binding protein (VDBP) rendering it incapable of being converted to the regulatory protein, Gc Macrophage Activating Factor (GcMAF). GcMAF is an immunologically important protein responsible for macrophage activation [17], thus Nagalase diminishes the body’s macrophage activating capacity, and elevated Nagalase has been reported in autoimmune disorders including systemic lupus erythematosus (SLE) [18].

We recently demonstrated that a cannabinoid receptor type 2 (CB2R) signalling was significantly upregulated in peripheral blood mononuclear cells (PBMCs) extracted from autistic children, suggesting that endocannabinoid (EC) system dysregulation could be involved in ASD-mediated immune impairments [19].

Using a new methodology of radiolabeling, the CB2R distribution was recently mapped using whole body positron emission tomography (PET) [20]. In healthy subjects without brain related pathology, the CB2R was demonstrated to map to the peripheral lymphoid immune system. Additionally, CB2R is expressed on both macrophages and microglial cells and activation of CB2R has been demonstrated to down-regulate ischemia-induced macrophage-microglial induced inflammation in an animal model [21].

The body produces arachidonate-based lipids, anandamide (N-arachidonoylethanolamide, AEA) and 2-arachidonoylglycerol (2-AG) which are binding ligands for the cannabinoid receptors [22]. CB2R appears to have primary immunomodulatory effects and CB2R-specific agonists and phytocannabinioids (for example, cannabidiol (CBD)) lack psychoactive properties [22].

GcMAF treatment seems to ameliorate autistic symptoms in some children [16]; however, the cellular and molecular pathways involved in the apparent therapeutic effect are not understood. We hypothesized that a potential therapeutic mechanism of GcMAF is related to transcriptional regulation of EC genes. We sought to investigate this mechanism *in vitro* using blood monocyte-derived macrophages (BMDMs) from autistic patients and controls.

## Materials and methods

### Subjects

We investigated 22 children with autism, and compared them to 20 age and sex matched healthy children used as a control group (age ranging 3 to 11 years; mean age: 7.06 ± 1.52 versus 7.38 ± 2.33 in autistic and healthy individuals, respectively). The subjects with autism were recruited into the study from the outpatient Biomedical Center for Autism Research and Treatment, Bari, Italy. Before entering the study, all of the children were administered the Autism Diagnostic Interview-Revised version [23], the Childhood Autism Rating Scales (CARS) [24], and the Autism Diagnostic Observation Schedule-Generic [25] to document the diagnosis of autism. All included patients met the *Diagnostic and Statistical Manual of Mental Disorders-IV* criteria for autism (DSM-IV-TR) [1]. In addition to meeting the criteria for autistic disorder, subject children were required to score at least 30 points on the CARS scale. Twenty healthy children were recruited among staff family members. Potential subjects were excluded if they had any of the following: a neurological or comorbid psychiatric disorder, epilepsy, history of liver, renal or endocrine disorders, current infection of any origin. Mental retardation or behavioral disorders, including Pervasive Developmental Disorder - Not Otherwise Specified (PDD-NOS), and inclusion criteria for attention deficit-hyperactivity disorder, were all considered exclusion criteria for control children. Children diagnosed with Asperger’s syndrome, fragile X syndrome and tuberous sclerosis were also excluded from the study. IQ test was not performed. Neither autistic subjects nor controls were receiving pharmacological interventions. Other exclusion criteria were celiac disease and/or other major diseases of the intestinal tract, such as inflammatory bowel disease or hepatic disorders.

Informed consent was obtained from the parents of all children enrolled in this study and assent was obtained from the healthy controls in compliance with Italian legislation and the Code of Ethical Principles for Medical Research Involving Human Subjects of the World Medical Association (Declaration of Helsinki).

### Isolation of peripheral blood mononuclear cells (PBMCs)

Mononuclear cells were extracted as previously described [9,19]. Briefly, less than 10 ml of fresh peripheral blood samples from autistic subjects and control donors were drawn and collected in sterile EDTA tubes (Becton Dickinson, Franklin Lakes, NJ, USA). Peripheral blood mononuclear cells (PMBCs) were isolated by centrifugation over Histopaque 1077 density gradient (Sigma Chemical, St Louis, MO, USA). Briefly, blood was diluted 1:1 in PBS (Sigma, St. Louis, MO, USA), overlaid onto lymphocyte separation media (Lymphocyte Separation Medium -Lonza, Walkersville, MD, USA), centrifuged at 2,200 rpm for 30 minutes at room temperature and plasma was removed. Mononuclear cell fraction was harvested and washed twice in PBS. The final pellet was re-suspended in RPMI 1640 complete medium (Lonza, Verviers, Belgium) containing 10% FBS (EuroClone-Celbio, Milan, Italy), 2 mM L-glutamine, 100 U/ml penicillin, and 100 mg/ml streptomycin (all Lonza, Verviers, Belgium) and incubated at 37°C with 5% CO_2_. Lymphocytes (non-adherent cells) were removed.

### Differentiation of macrophages from PBMCs

**Figure 1:**
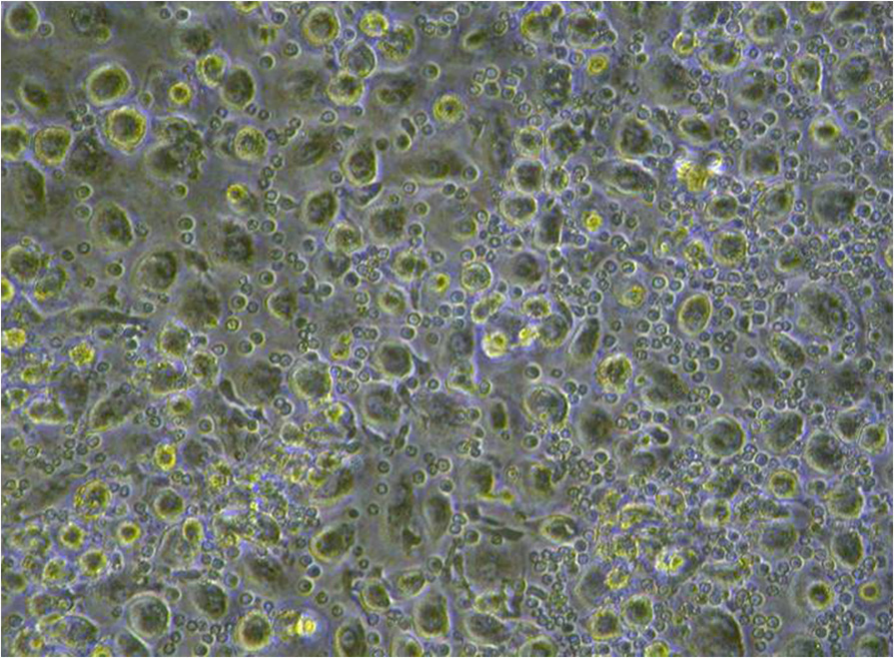
**Representative optical photomicrograph of blood monocyte-derived macrophages (BMDMs) *in vitro *** **culture from autistic children.**

In order to obtain fully differentiated human blood monocyte-derived macrophages (BMDMs), PBMCs were then cultured for about ten days in the presence of 25 ng/mL recombinant human macrophage colony-stimulating factor (Peprotech, London, UK) [26,27] (Figure 1).

### *in vitro* treatment

GcMAF was added at 0.1 ng/ml final concentration to BMDMs from healthy control and autistic patients. The dose of GcMAF was chosen on the basis of a previous work demonstrating that maximal stimulation of PBMCs was achieved with 0.1 ng/ml [28]. GcMAF was kindly provided by Immuno Biotech (St. Peter Port, Guernsey, UK). Following incubation of the cells for 24 hours in the presence of GcMAF, some cells were lysed for the extraction and analysis of RNA (Reverse Transcriptase-Polymerase Chain Reaction (RT-PCR)), and proteins via Western blot analysis; alternatively, other cells were fixed for fluorescence-based immunocytochemistry analysis. Comparisons between pre-post treatment levels of RNA and protein expression were made for both autistic individuals and healthy controls.

### RNA extraction and RT-PCR

The RNA was extracted from BMDMs using a RNA Tri-Reagent (Molecular Research Center Inc., Cincinnati, OH, USA) according to the manufacturer’s protocol. The total RNA concentration and integrity were determined by Nanodrop® ND-1000 UV spectrophotometer (Nano-Drop® Technologies, Thermo Scientific, Wilmington, DE, USA). The mRNA levels of the EC genes under analysis were measured by RT-PCR amplification, as previously reported [19]. Reverse Transcriptase from Avian Myeloblastosis Virus (AMV-RT; Promega, Madison, WI, USA) was used. For first-strand cDNA synthesis, 200 ng total RNA, random hexamers, dNTPs (Promega, Madison, WI, USA), AMV buffer, AMV-RT and recombinant RNasinribonuclease inhibitor (Promega, Madison, WI, USA) were assembled in diethyl-pyrocarbonate-treated water to a 20 μl final volume and incubated for ten minutes at 65°C and one hour at 42°C. RT minus controls were carried out to check potential genomic DNA contamination. These RT minus controls were performed without using the reverse transcriptase enzyme in the reaction mix. Aliquots of 2 μl cDNA were transferred into a 25 μl PCR reaction mixture containing dNTPs, MgCl_2_, reaction buffer, specific primers and GoTaq Flexi DNA polymerase (Promega, Madison, WI, USA), and amplification reactions using specific primers and conditions for human genes under analysis were carried out. Sequences for the human mRNAs from GeneBank (DNASTAR Inc., Madison, WI, USA) were used to design specific primer pairs for RT-PCRs (OLIGO 4.05 software, National Biosciences Inc., Plymouth, MN, USA) (Table 1) [19]. Each RT-PCR was repeated at least three times to achieve the best reproducibility data. The levels of mRNA measured were normalized with respect to glyceraldehyde-3-phosphate dehydrogenase (*GAPDH*), which was chosen as the housekeeping gene. Indeed *GAPDH* is one of the most stably expressed genes in human peripheral blood [29]. To our knowledge, there is no molecular evidence of variation in *GAPDH* mRNAlevels in autism disorders [19]. The gene expression values were expressed as arbitrary units ± SEM. Amplification of the genes of interest and *GAPDH* was performed simultaneously. PCR products were resolved into 2% agarose gel. A semi-quantitative analysis of mRNA levels was carried out by the Gel Doc EZ UV System (Bio-Rad, Hercules, CA, USA).

**Table 1:** Primer sequences, annealing temperatures, and product sizes for RT-PCRs

** Gene**	** Sense primer (5′-3′)**	** Antisense primer (5′-3′)**	**Annealing temperature (°C)**	**Product sizes (bp)**
*CB2R*	TTGGCAGCGTGACTATGACC	AGGAAGGCGATGAACAGGAG	55	274
*FAAH*	GGCCACACCTTCCTACAGAA	GTTTTGCGGTACACCTCGAT	58	218
*NAPE-PLD*	GAAGCTGGCTTAAGAGTCAC	CCGCATCTATTGGAGGGAGT	60	178
*GAPDH*	TCACCAGGGCTGCTTTTAAC	GGACTCCACGACGTACTCAG	55	242

PCR primers were designed by using the computer program OLIGO 4.05 software (National Biosciences Inc., Plymouth, MN, USA) and were purchased from PRIMM (Milan, Italy).

### Protein extraction and Western blot analysis

For protein extraction, BMDMs were suspended in protein lysis buffer (HEPES 25 mM; EDTA 5 mM; SDS 1%; Triton X-100 1%; PMSF 1 mM; MgCl_2_ 5 mM; Protease Inhibitor Cocktail (Roche, Mannheim, Germany); Phosphatase Inhibitor Cocktail (Roche, Mannheim, Germany)). Protein concentration was determined using the method described by Bradford [30]. For CB2R detection, each sample was loaded, electrophoresed in a 15% SDS-polyacrylamide gel and electroblotted onto a nitrocellulose membrane. The membrane was blocked in 5% milk, 1X Tris-buffered saline and 0.05% Tween-20. Primary antibodies to detect CB2R (Calbiochem-Merck, Darmstadt, Germany) were used according to the manufacturer’s instructions at 1:250 dilutions [19]. The rabbit anti-CB2R antibody detects endogenous levels of the human 45 kDa fragment of CB2R protein. The antibody does not cross-react with the CB1 receptor protein and, according to the manufacturer, was validated with a recombinant protein consisting of the first 33 amino acids of human CB2R used as a positive control. For mannose receptor detection, each sample was loaded, electrophoresed in a precast gradient 4 to 12% SDS-polyacrylamide gel using Bolt® system (Life Technologies, Monza, Italy) and electroblotted onto a nitrocellulose membrane. The membrane was blocked in 5% milk, 1X Tris-buffered saline and 0.05% Tween-20. Primary antibodies to detect mannose receptor (ab64693 Abcam, Cambridge, UK) were used according to the manufacturer’s instructions at 1:1,000 dilutions. Immunoreactive signals were detected with a horseradish peroxidase-conjugated secondary antibody and reacted with an ECL system (Amersham Pharmacia, Uppsala, Sweden). To assess equal loading, protein levels were normalized with respect to the signal obtained with Coomassie Blue staining, as previously reported [31]. We used Coomassie Blue staining as equal loading control as this method overcomes the possibility that housekeeping proteins could vary in this pathology or be saturated at the levels of loading [19]. However, we confirmed the signals obtained by Coomassie Blue staining with respect to the signal obtained with anti-β-tubulin monoclonal antibodies (A2066 Sigma Chemical, St Louis, MO, USA; 1:1,000 dilution). The semi-quantitative analysis of protein levels was carried out by the ChemiDoc-It 5000, using VisionWorks Life Science Image Acquisition and Analysis software (UVP, Upland, CA, USA).

### Immunocytochemistry

For immunocytochemical analysis, BMDMs were re-suspended at 1x10^6^ cell/mL in RPMI 1640 complete medium (Lonza, Verviers, Belgium) containing 10% FBS (EuroClone-Celbio, Milan, Italy), 2 mM L-glutamine, 100 U/ml penicillin, and 100 mg/ml streptomycin (all Lonza, Verviers, Belgium), were plated on slides with a 12-well plate and incubated at 37°C with 5% CO_2_. Cells were then fixed with 4% paraformaldehyde fixative. After washing in PBS, non-specific antibody binding was inhibited by incubation for 30 minutes in blocking solution (1% BSA in PBS). Primary antibodies were diluted in PBS blocking buffer and slides were incubated overnight at 4°C in primary antibodies to human Notch (1:100; Santa Cruz Biotechnology, Santa Cruz, CA, USA) or to human Ki67 proliferation marker (1:200; Santa Cruz Biotechnology, Santa Cruz, CA, USA). Fluorescent-labeled secondary antibodies (1:1,000; Alexa Fluor 488, Molecular Probe; Invitrogen, Carlsbad, CA, USA) specific to the IgG species used as a primary antibody were used to locate the specific antigens in each slide. Cells were counterstained with bisbenzimide (Hoechst 33258; Hoechst, Frankfurt, Germany) and mounted with mounting medium (90% glycerol in PBS). Fluorescently-labeled slides were viewed with a fluorescence microscope (Leica, Wetzlar, Germany) and with a fluorescence confocal microscope (LSM 710, Zeiss, Oberkochen, Germany). Immunofluorescence images were analyzed with Leica FW4000 software (Leica, Wetzlar, Germany) and with Zen Zeiss software (Zeiss, Oberkochen, Germany). Quantification of Ki67-ir profiles was performed by an observer blind to the treatment. Cell positive profile quantification was performed on each digitized image, and the reported data are the intensity means ± SE on counterstained cells per group. Only bisbenzimide counterstained cells were considered as positive profiles so as to avoid overcounting cells.

### Statistical analysis

Biomolecular data are expressed as means ± SEM. ANOVA, followed by Student-Neuman-Keuls *post hoc* test, was used to determine the statistical significance among groups. *P* < 0.05 was considered statistically significant.

## Results

### GcMAF was able to normalize endocannabinoid system gene dysregulation in blood monocyte-derived macrophages (BMDMs) in autistic children

As we already demonstrated [19], studying EC system gene expression mainly by RT-PCR is far more sensitive for the detection of gene expression than immunocytochemistry [9,32]. We evaluated the GcMAF effects on *NAPE-PLD* (N-acyl phosphatidylethanolamine phospholipase D), a protein-coding gene [GC07M102742]. The gene codes for the enzyme which hydrolyzes N-acyl-phosphatidylethanolamines (NAPEs) to produce N-acylethanolamines (NAEs) and phosphatidic acid and, specifically, the generation of anandamide (N-arachidonoylethanolamine), the ligand of cannabinoid receptors. When compared to healthy controls, the semiquantitative analysis of BMDM-extracted mRNA levels, measured by RT-PCR amplification, showed an increase in the *NAPE-PLD* gene in BMDMs of autistic patients (mean ± SE of arbitrary units: 1.20 ± 0.34 versus 0.71 ± 0.11, *P* < 0.05, in BMDMs from autistic children and healthy subjects, respectively).

**Figure 2:**
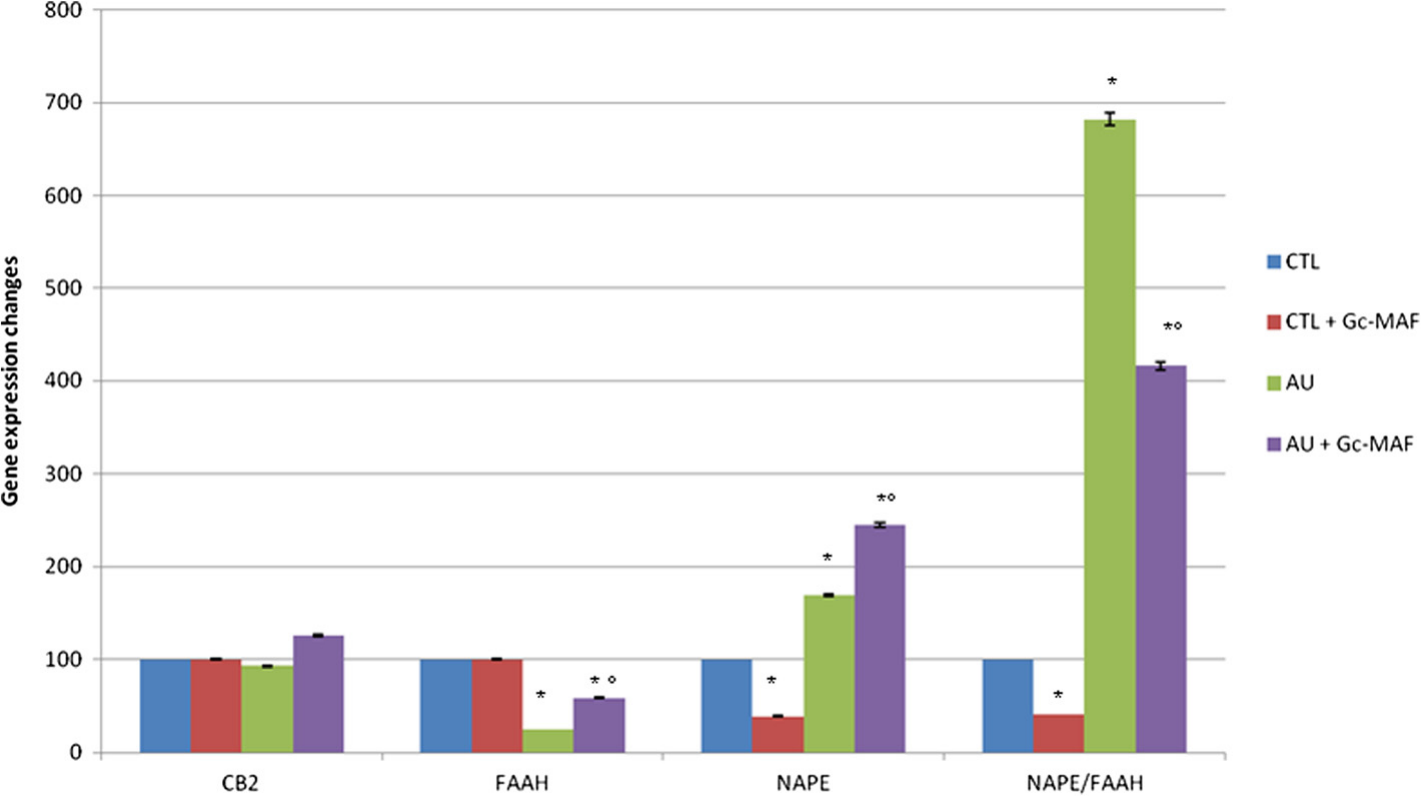
**Expression of the enzymes NAPE-PLD and FAAH, and *CB2R *genes in blood monocyte-derived macrophages (BMDMs).** The ratio NAPE/FAAH was also reported. The measured mRNA levels were normalized with respect to *GAPDH* (housekeeping gene) and gene expression values were expressed as a percentage of arbitrary units ± SEM. * indicates significant difference versushealthy controls; *°* indicates significant difference versus GcMAF untreated autistic BMDMs. *P-*values <0.05 were considered statistically significant. CTL, healthy control subjects;AU, autistic patients. Values were reported in percentage versus healthy control values.

We also evaluated the gene for fatty acid amide hydrolase, (*FAAH*) [GC01P046860]. FAAH is a membrane associated enzyme which hydrolyzes bioactive amides, including the EC, anandamide. We observed that the mRNA levels of the *FAAH* enzyme gene were decreased (mean ± SE of arbitrary units: 0.40 ± 0.08 versus 1.60 ± 0.06, *P* < 0.05, in BMDMs from autistic children and healthy subjects, respectively); the NAPE-PLD/FAAH ratio was significantly increased (mean ± SE of arbitrary units: 3.00 ± 0.84 versus 0.44 ± 0.07 in BMDMs from autistic children compared to healthy subjects, respectively); mRNA levels of *CB2R* gene (mean ± SE of arbitrary units: 0.40 ± 0.01 versus 0.43 ± 0.02, *P* > 0.05, in BMDMs from autistic children and healthy subjects, respectively) were not changed (Figure 2).

In BMDMs of autistic children, GcMAF treatment was able to significantly increase gene expressions both NAPE-PLD (mean ± SE of arbitrary units: 1.20 ± 0.34 and 1.74 ± 0.18, *P* < 0.05, before and after GcMAF treatment in autistic BMDMs, respectively) and FAAH (mean ± SE of arbitrary units: 0.40 ± 0.08 versus 0.95 ± 0.04, *P* < 0.05, before and after GcMAF treatment in autistic BMDMs, respectively), whereas the NAPE-PLD/FAAH ratio was significantly reduced (mean ± SE of arbitrary units: 3.00 ± 0.84 versus 1.83 ± 0.19, before and after GcMAF treatment in autistic BMDMs, respectively). The mRNA levels of *CB2R* gene were not affected by GcMAF treatment (mean ± SE of arbitrary units: 0.40 ± 0.01 versus 0.54 ± 0.01, *P* > 0.05, before and after GcMAF treatment in autistic BMDMs, respectively). No changes were observed in GcMAF treated BMDMs of healthy control children with respect to untreated BMDMs, except for a slight decrease in *NAPE-PLD* gene expression (not affecting NAPE-PLD/FAAH ratio) (Figure 2).

### GcMAF affected CB2R protein levels in BMDMs

GcMAF was also able to reduce the protein levels for CB2R in BMDMs from autistic children with respect to treated BMDMs from healthy controls. Since CB2Rs are G protein-coupled receptors, they show post-translational regulation [33]. We therefore determined the protein levels of CB2R by Western blot analysis.

**Figure 3:**
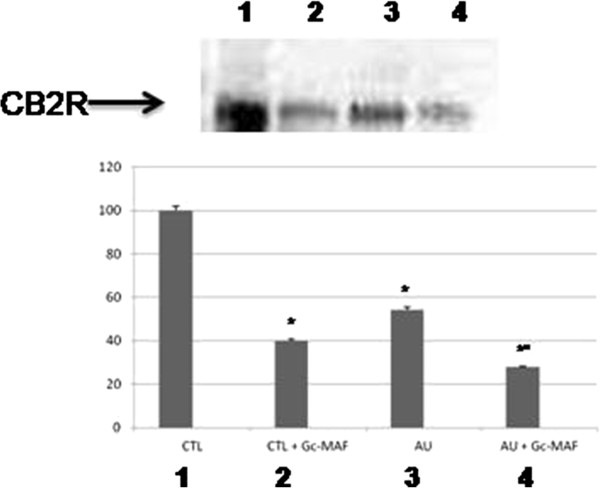
**Representative Western blot analysis of CB2R protein levels in the blood monocyte-derived macrophages (BMDMs) obtained from the autistic children and the healthy controls. **(1) untreated BMDMs from healthy control subjects; (2) GcMAF-treated BMDMs from healthy control subjects; (3) untreated BMDMs from autistic disorder subjects; (4) Gc-MAF-treated BMDMs from autistic disorder subjects. The histograms indicate percentage variations in CB2R protein levels in the BMDMs. * indicates significant difference versus healthy controls; *°* indicates significant difference versusGcMAF untreated autistic BMDMs.

Western blot analysis showed a strong decrease in CB2R protein levels in GcMAF treated BMDMs from autistic children as compared to untreated macrophages (mean ± SE of arbitrary units: 3.24 ± 0.54 versus 1.66 ± 0.39, *P* < 0.05, before and after GcMAF treatment in autistic BMDMs, respectively). Interestingly, GcMAF was also able to decrease CB2R protein levels in GcMAF treated BMDMs from healthy controls as compared to untreated BMDMs (Figure 3) (mean ± SE of arbitrary units: 5.97 ± 0.34 versus 2.39 ± 0.43, *P* < 0.05, before and after GcMAF treatment in healthy control BMDMs, respectively).

The difference between the unchanged *CB2R* mRNA levels and the decrease in CB2R protein levels in autistic GcMAF treated cells is not surprising, as we have already shown [19]. Indeed, protein levels and functions are affected by post-translational control. The levels of CB2R in the cell are strictly regulated in a multilevel system of regulation [34], and there is not a direct correlation between mRNA transcripts and protein levels [19].

### GcMAF was able to trigger overall macrophage deactivation in autistic samples

**Figure 4:**
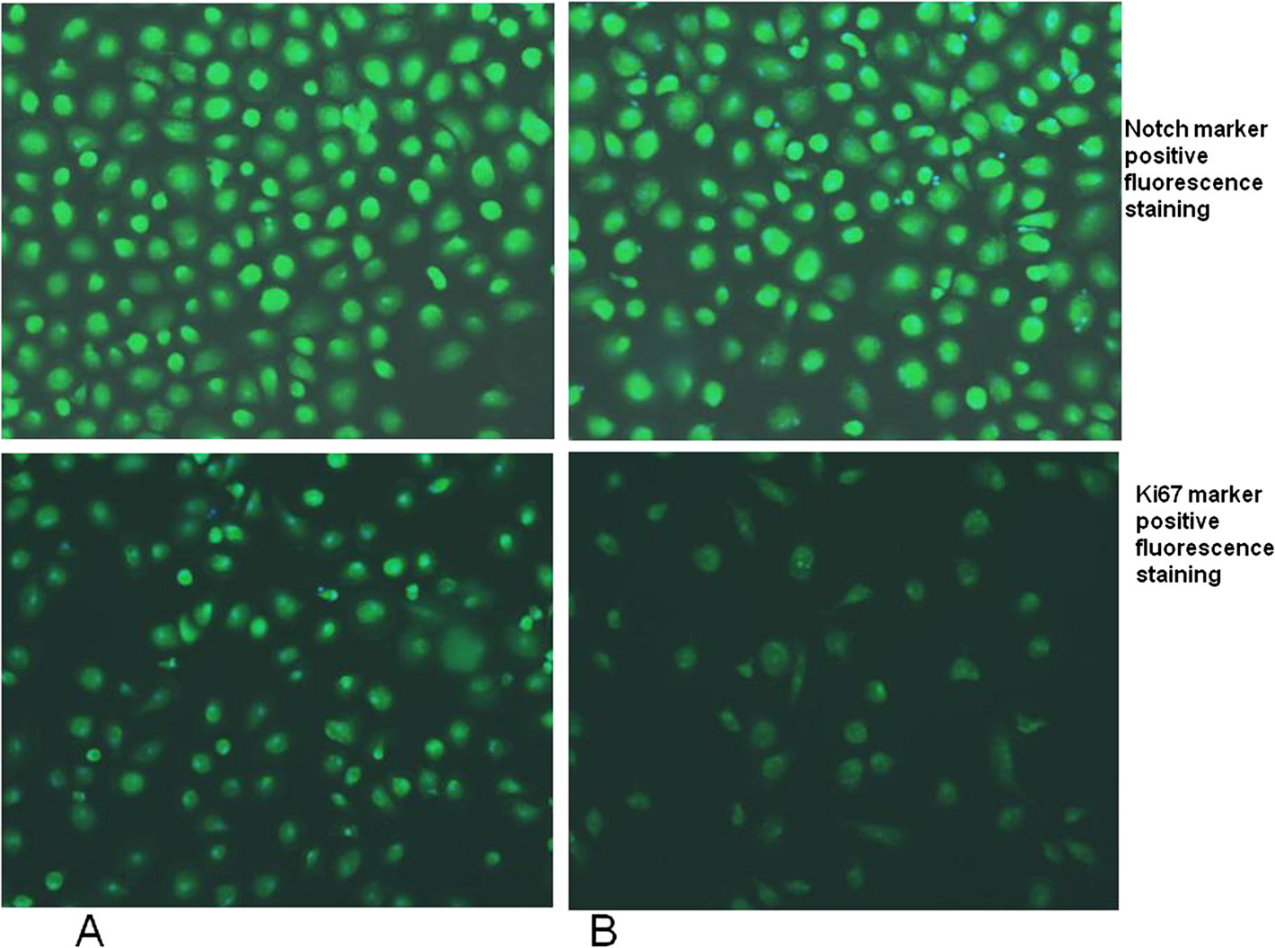
**Representative fluorescent photomicrograph of blood monocyte-derived macrophages (BMDMs) from autistic patients showing immunocytochemistry (green fluorescent) for Notch (top) and Ki67 markers (bottom).** To correctly identify cells, their nuclei were counterstained with bisbenzimide (blue fluorescence). **(A)** untreated BMDMs; **(B)** GcMAF treated BMDMs.

In order to check cellular activation, fluorescence-based immunocytochemical analysis on macrophage cell culture was performed. In detail, Notch staining was early carried out. Notch is a protein mainly involved in stem cell maintenance, cell differentiation and cellular homeostasis regulation [35]; however, Notch signaling pathway was reported in activated pro-inflammatory macrophages and it is involved in regulating the expression of il12p40 [36]. Recently, it has been proposed a putative role of Notch signaling in autism [37]. We did not find any changes in Notch immunostaining profiles in GcMAF treated blood monocyte-derived macrophages from autistic children as compared to untreated macrophage cells, as analyzed by fluorescence microscopy (Figure 4).

**Figure 5:**
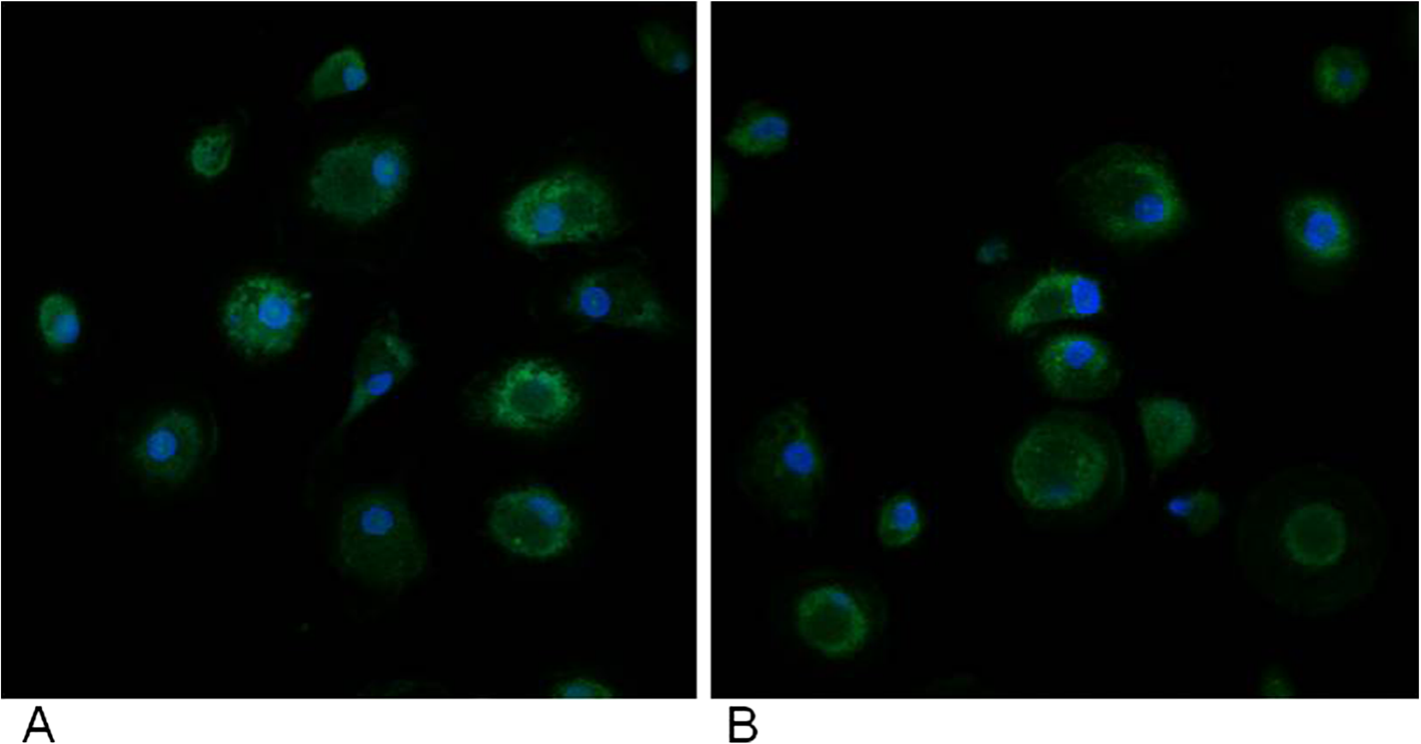
**Representative quantitative fluorescent confocal-photomicrograph of blood monocyte-derived macrophages (BMDMs) from autistic patients showing immunocytochemistry (green fluorescent) for Ki67 marker.** To correctly identify cells, their nuclei were counterstained with bisbenzimide (blue fluorescence). **(A)** untreated BMDMs; **(B)** Gc-MAF treated BMDMs.

As macrophages possess proliferation capacity [38], we investigated the effect of GcMAF on this cellular activity through Ki67 proliferation marker immunostaining. Interestingly, GcMAF treatment showed reduction in the immunostaining profiles of the proliferation marker Ki67 in GcMAF treated monocyte-derived macrophages from autistic children, as compared to untreated macrophage cells (Figure 4). We quantified this reduction through confocal fluorescence microscopy, showing a decrease of 23% in GcMAF treated monocyte-derived macrophages from autistic children as compared to untreated macrophage cells (mean ± SE of arbitrary units: 0.32 ± 0.04 versus 0.24 ± 0.06, *P* < 0.05, before and after GcMAF treatment in autistic BMDMs, respectively) (Figure 5). GcMAF was also able to reduce Ki67 immunostaining in BMDMs from healthy controls, as compared to untreated macrophage cells (mean ± SE of arbitrary units: 0.17 ± 0.01 versus 0.012 ± 0.003, *P* < 0.05, before and after GcMAF treatment in healthy control BMDMs, respectively) (picture not shown). It is noteworthy to consider that BMDMs from autistic children were more activated than BMDMs from healthy controls (mean ± SE of arbitrary units: 0.32 ± 0.04 versus 0.17 ± 0.01, *P* < 0.05, in BMDMs from autistic children and healthy subjects, respectively).

### GcMAF was able to reduce the protein levels of the alternative activated phenotype M2 macrophage marker

**Figure 6:**
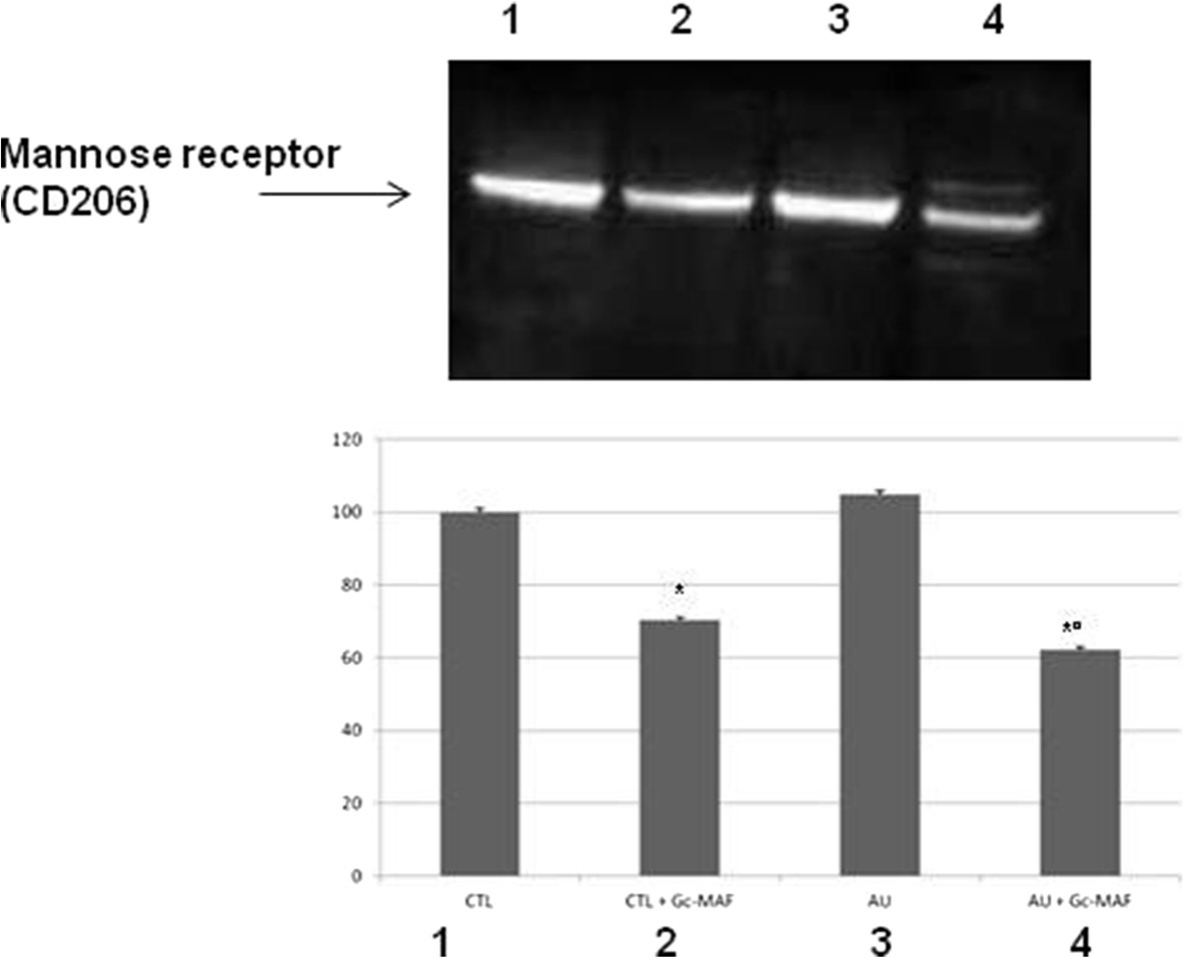
**Representative Western blot analysis of mannose receptor protein levels in the blood monocyte-derived macrophages (BMDMs) obtained from the autistic children and the healthy controls.** (1) untreated BMDMs from healthy control subjects; (2) GcMAF-treated BMDMs from healthy control subjects; (3) untreated BMDMs from autistic disorder subjects; (4) GcMAF-treated BMDMs from autistic disorder subjects. The histograms indicate percentage variations in mannose receptor protein levels in the BMDMs. * indicates significant difference versus healthy controls; *°* indicates significant difference versus GcMAF untreated autistic BMDMs.

To further investigate the GcMAF effect in specific cellular activation in macrophages, we quantified the protein levels of mannose receptor by Western blot analysis. The macrophage mannose receptor (alternative name CD206) mediates the endocytosis of glycoproteins by macrophages and it is considered a specific marker for alternative activated phenotype M2 macrophages [39,40]. Western blot analysis showed a decrease in mannose receptor protein levels in GcMAF treated BMDMs from autistic children as compared to untreated macrophages (mean ± SE of arbitrary units: 28.1 ± 0.41 versus 16.7 ± 0.94, *P* < 0.05, before and after Gc-MAF treatment in autistic BMDMs, respectively) (Figure 6). GcMAF was also able to decrease mannose receptor protein levels in GcMAF treated BMDMs from healthy controls as compared to untreated BMDMs (mean ± SE of arbitrary units: 26.8 ± 0.21 versus 18.9 ± 0.47, *P* < 0.05, before and after GcMAF treatment in healthy control BMDMs, respectively) (Figure 6).

## Discussion

In this study, we demonstrated for the first time a cannabinoid system mediated biomolecular mechanism and cellular effect of GcMAF in cultured BMDMs from autistic subjects.

Anti-cancer effects of GcMAF have been described since Yamamoto, *et al*., [41] first demonstrated the vitamin D binding protein macrophage activating effects and the linkage to specific glycosylation of the Gc precursor protein [41]. It has been proposed that GcMAF possesses tumor killing activity through the activation of macrophages [42]. GcMAF-activated macrophages are indeed able to recognize the tumor cell surface abnormalities through a considerable variation of their receptors: in this way, they exert one potent tumoricidal effect [43].

Kanda *et al*., [44] first described another anti-tumor effect of GcMAF related to its inhibition of angiogenesis, presumably mediated through the CD36 receptor, while Solinas *et al*., [45] observed the non-psychoactive CB2R-binding cannabinoid, CBD also inhibited neoangiogenesis. Similarly, calcitriol (1,25-dihydroxyvitamin D3), also exerts anti-angiogenesis effects, creating an interesting potential nexus between vitamin D, GcMAF, and the EC system in the etiology and pathology of numerous immune-mediated disorders, including autism [46,47].

In relation to autism, the vitamin D deficiency hypothesis has been extensively investigated, with prenatal and/or early postnatal vitamin D deficiency demonstrating increased risks for development [48,49]. Autism is now considered a multifactorial disease associated with complex genetic and environmental interactions contributing to various risk factors [2]. Moreover, dietary vitamin D seems to be involved in complex epigenetic events. Vitamin D, via its ligand-activated nuclear hormone receptor, is involved in the regulation of pro-inflammatory genes, as well as key cellular events [2]. Nagalase activity is increased in the serum of autistic children [16]. As we previously mentioned, Nagalase is the enzyme responsible for deglycosylation of the vitamin D-binding protein (VDBP), also known as Gc-globulin (group-specific component). Gc-globulin is the precursor of GcMAF, so Nagalase interferes with macrophage regulation by reducing GcMAF production [50]. A predictable consequence of increased Nagalase activity in the serum of children with autism is, therefore, immunosuppression in a way similar to its observed effects in autoimmune patients, for example, SLE [18].

It has been demonstrated that in PBMCs, GcMAF is able to increase the production of the second messenger cyclic AMP [28]. These data, when combined with our current observations demonstrating GcMAF normalizes EC gene expression, enhance the hypothesis of a potential action of GcMAF on the EC system. Indeed, the EC system is based on receptors coupled to G (i/o) proteins, which are associated with inhibition of cyclic AMP formation [51]. In BMDMs from autistic children, we evaluated whether the involvement of EC signalling could drive a decrease of cyclic AMP. We found an increase in AEA-biosynthetic enzyme NAPE-PLD, together with a decrease in the AEA catabolic enzyme FAAH expressions, indicating an overall increase in the EC AEA levels. AEA is a natural agonist of CB2R and down-regulates cyclic AMP production. Agonist-induced inhibition of adenylyl cyclase in cells expressing human CB2Rs has been demonstrated [52]. Our findings support the influence of GcMAF on the EC system which may result in normalized cycling AMP activity.

Our current findings further support the involvement of the EC system in autism associated immunological disruptions. We previously found that CB2Rs were strongly up-regulated in PBMCs from autistic children [19]. The observations in this present study agree with our hypothesis that the EC system in autism orchestrates the apparent nexus of the peripheral and central neuro-immunologically mediated effects in autism. Interestingly, while in our previous work, the CB2R was over-activated in PBMCs from autistic children, in the present study, we found a decrease in CB2R protein levels in BMDMs from autistic patients. Taking in account the difference between the two-cell systems, as BMDM cells are derived, through differentiation, from PBMCs, this result could indicate a dual role of the CB2R: activation in monocytes to trigger immune imbalance, and deactivation in differentiated macrophages to further persist in the immune dysregulation [19].

The fact that GcMAF was able to reduce Ki67 proliferation marker staining together with a decrease in CD206 positive profiles in BMDMs is not surprising. Indeed, it has been demonstrated that macrophages are altered in autism and this pathology is accompanied by an activation of the macrophages, together with immune alterations and pro-inflammatory cytokines (that is IL-1β) over-production [53,54]. Specifically, Al-Ayadhi and Mostafa [55] found macrophage-derived chemokine (MDC) and thymus and activation-regulated chemokine (TARC), both were significantly elevated in ASD serum and further demonstrated the level of elevation of both markers directly correlated with the severity of autism [55]. Molloy *et al*., (2006) also demonstrated a predominately Th2 cytokine shift in the serum of children with autism [56]. In this study, our findings of pre/post Ki67 and CD206 are in agreement with the Th2/M2 macrophage observations and of a tendency toward autoimmunity. Macrophages are not static and can readily shift from immature forms and between M1 and M2 states depending on the local tissue signalling [57].

Recently, a different cohort of Italian children was assessed for anti-brain antibodies [58]. In that research, the presence of specific anti-brain antibody profiles was associated with the severity of cognitive impairment in autism.

The potential for either commensal or pathogenic microbes to trigger immune dysregulation and autoimmunity with resultant neuropsychiatric symptoms was recently reviewed by Hornig [59]. The CB2R profile observed in this study is consistent with these mechanisms and could be tied to either infection or alteration of the gut microbiome as illustrated in a mouse model of autism [60]. In that murine study, alteration in short chain fatty acids as a consequence of valproate exposure, modelled autistic characteristic in the mice.

EC are derived from dietary fatty acids. Several studies illustrate the effect of diet on blood, tissue and brain EC levels [61,62]. So, the measured differences in this study in the CB2R may be the consequence of dietary and/or microbiome changes in the autism population when compared to the controls.

## Conclusions

This study demonstrates a biomolecular effect of GcMAF in BMDMs from autistic patients, providing further evidence for a positive use of this molecule in autism treatment. It also seems likely that the CB2R is a potential therapeutic target for ASD interventions. These initial findings will require further study in order to better elucidate the molecular pathways involved in GcMAF effects.

## Abbreviations

ASDs: autism spectrum disorder; AEA: N-arachidonoylethanolamide; 2-AG: 2-arachidonoylglycerol; BMDMs: blood monocyte-derived macrophages; CBD: cannabidiol; CB2R: cannabinoid receptor type 2; DSM-IV-TR: Diagnostic and Statistical Manual of Mental Disorders-IV criteria for autism; EC: endocannabinoid; FAAH: fatty acid amide hydrolase; FBS: fetal bovine serum; NK: natural killer; GAPDH: glyceraldehyde-3-phosphate dehydrogenase; GcMAF: Gc Macrophage Activating Factor; IL: interleukin; NAPE-PLD: N-acyl phosphatidylethanolamine phospholipase D; PET: positron emission tomography; PBMCs: peripheral blood mononuclear cells; PBS: Phosphate buffer saline; PDD-NOS: Pervasive Developmental Disorder - Not Otherwise Specified; RT-PCR: Reverse Transcriptase-Polymerase Chain Reaction; RPMI: Roswell Park Memorial Institute; SLE: systemic lupus erythematosus; VDBP: vitamin D3 binding protein.

## Competing interests

The authors declare that they have no competing interests.

## Authors’ contributions

DS designed the study, carried out the biochemical and immunocytochemical experiments, performed the statistical analysis and wrote the manuscript. JJB participated in the design of the study, wrote the manuscript and edited English language. AC carried out the biomolecular experiments. NA conceived of the study, participated in its design and provided funding support. All authors read and approved the final manuscript.
